# Enhancing UCSF Chimera through web services

**DOI:** 10.1093/nar/gku377

**Published:** 2014-05-26

**Authors:** Conrad C. Huang, Elaine C. Meng, John H. Morris, Eric F. Pettersen, Thomas E. Ferrin

**Affiliations:** Resource for Biocomputing, Visualization, and Informatics, Department of Pharmaceutical Chemistry, University of California San Francisco, San Francisco, CA 94143, USA

## Abstract

Integrating access to web services with desktop applications allows for an expanded set of application features, including performing computationally intensive tasks and convenient searches of databases. We describe how we have enhanced UCSF Chimera (http://www.rbvi.ucsf.edu/chimera/), a program for the interactive visualization and analysis of molecular structures and related data, through the addition of several web services (http://www.rbvi.ucsf.edu/chimera/docs/webservices.html). By streamlining access to web services, including the entire job submission, monitoring and retrieval process, Chimera makes it simpler for users to focus on their science projects rather than data manipulation. Chimera uses Opal, a toolkit for wrapping scientific applications as web services, to provide scalable and transparent access to several popular software packages. We illustrate Chimera's use of web services with an example workflow that interleaves use of these services with interactive manipulation of molecular sequences and structures, and we provide an example Python program to demonstrate how easily Opal-based web services can be accessed from within an application. Web server availability: http://webservices.rbvi.ucsf.edu/opal2/dashboard?command=serviceList.

## INTRODUCTION

UCSF Chimera (1–3) is a program for the interactive visualization and analysis of molecular structures and related data, designed for use by structural biologists, biomedical researchers and others interested in molecular structure and function. Chimera is a desktop application with roots primarily in interactive visual analysis, but advances in desktop performance and the advent of web services have allowed both augmenting its visualization capabilities and expanding its set of features to include more computationally intensive tasks.

To support the visualization of known structures, Chimera took advantage of internet-based services early in its development, initially focusing on the use of internet services to fetch data from data repositories such as the PDB (4), NDB (5), EMDB (6), etc. (http://www.rbvi.ucsf.edu/chimera/docs/webservices.html#data). Recently, however, focus has been on utilizing internet (web) services to extend the analytical capabilities we provide to understand the structure of biological molecules, how those molecules interact and the biological implications of those interactions (http://www.rbvi.ucsf.edu/chimera/docs/webservices.html#comp). Two key enablers for the integration of web services into Chimera were the use of an existing broadly deployed web service framework, Opal (7), and the creation of an interactive tool within Chimera that manages external computations running asynchronously, including those utilizing external web services.

Having both enablers allows us to easily deploy a new web service and utilize that web service from within the Chimera application. However, there is still significant effort required to develop a user interface for specifying the data to provide to the web service and to add results returned by the service into the visualization. When integrating a new, external computational tool, there are three factors that are taken into account.

- Does this tool augment or integrate with existing capabilities? Chimera is not designed to provide solutions for every aspect of biology. The core of Chimera is the visualization of biological structures and related data. When a web service uses or produces data that Chimera can already handle, it becomes a good candidate for integration because existing code can be reused. For example, we added interfaces to multiple sequence alignment (MSA) services such as Clustal Omega (8) and MUSCLE (9) because we can use Chimera's existing Multalign Viewer (MAV) tool (2) to analyze their output; the integration consists of (a) constructing the user interface for selecting the input sequences, (b) invoking the web service and (c) displaying the resulting alignment in MAV. Leveraging existing tools makes it easy to incorporate web service functionality with minimal programming efforts.
- Does this tool interfere with the interactive use of Chimera? It is critical that the interactive nature of the environment not be severely impacted by new capabilities. The development of support for asynchronous tasks allows us to externalize computation (either on the local workstation or through web services) to avoid negatively impacting the user experience. When possible, the computation takes place concurrently with the visualization session, with the results being displayed as they become available.
- Should this tool run locally or through a web service? If a computation is very fast, it may make sense to run it locally to avoid the performance overhead of sending data to an external server. It might also make sense to run computations locally if the size of the data set is extremely large (for example, large volumetric data). On the other hand, if the service utilizes a large repository of data [e.g. all Protein Data Bank (PDB) entries] that would be more efficient to maintain centrally, it makes sense to utilize an external web service. Chimera offers the option to run a service locally if the required application is installed on the local computer.

The full list of computational web services utilized by Chimera is maintained at: http://www.rbvi.ucsf.edu/chimera/docs/webservices.html#comp. Table 1 is a selected list of computational web services utilized by Chimera that are provided by the Resource for Biocomputing, Visualization, and Informatics (RBVI) and by the National Biomedical Computation Resource (NBCR), both of which are National Institutes of Health Biomedical Technology Research Centers. Besides the sequence alignment services mentioned above, these include BLAST sequence searching (10), Modeller comparative modeling (11,12), calculation of theoretical small-angle X-ray scattering profiles (13), APBS electrostatics calculations (14), PDB2PQR preparation of structures for electrostatics calculations (14,15), and AutoDock Vina molecular docking (16).

**Table 1.** Partial list of computational web services used by Chimera

Service	Description	Host
BlastProtein	Search for similar protein sequences using BLAST (10) in PDB, nr or uniref database.	RBVI
Clustal Omega	Perform MSA for protein or DNA/RNA using Clustal Omega (8).	RBVI
MUSCLE	Perform MSA for protein or DNA/RNA using MUSCLE (9).	RBVI
Modeller9v8	Perform comparative protein structure modeling using Modeller (11,12).	RBVI
SAXS2.1	Compute theoretical x-ray scattering profile of a structure and fitting of experimental profile (13).	RBVI
APBS 1.3	Compute electrostatic interactions between molecular solutes in salty, aqueous media (14).	NBCR
PDB2PQR 1.8	Prepare molecular structures for continuum electrostatics calculations (14,15)	NBCR
AutoDock Vina 1.1.2	Perform molecular docking between ligand and receptor molecules (16).	NBCR

To get a sense of how computational web services are integrated into Chimera, we describe an example workflow highlighting the interaction of web services with the molecular visualization and analysis tools provided internally by Chimera. The subsequent section describes details of how Chimera interacts with these web services and the web service infrastructure.

## EXAMPLE WORKFLOW WITH CHIMERA AND WEB SERVICES

Several common workflows interleave use of web services with interactive manipulation of structures and other data in Chimera. A frequent use case is comparative (homology) modeling of some target protein to facilitate functional annotation or inhibitor design. The search for modeling templates and the modeling calculation itself can both use RBVI web services, and further web services may also be involved, depending on the project. The features described below are available in Chimera version 1.9; the user is not required to install any other software packages. More detailed, step-by-step tutorials on comparative modeling with Chimera can be viewed online: http://www.rbvi.ucsf.edu/chimera/docs/UsersGuide/tutorials/dor.html, http://www.cgl.ucsf.edu/chimera/videodoc/Modeller/.

As an example, we will model the structure of an uncharacterized solute-binding protein from *Roseobacter denitrificans* [UniProt (17) Q16BX4, Enzyme Functional Initiative (18) target EFI-510232]. Bacterial solute-binding proteins recognize specific small molecules in the periplasmic space with a ‘Venus flytrap mechanism’ (19) and transfer them to cognate channel proteins for transport across the membrane into the cytoplasm.

**Figure 1.**
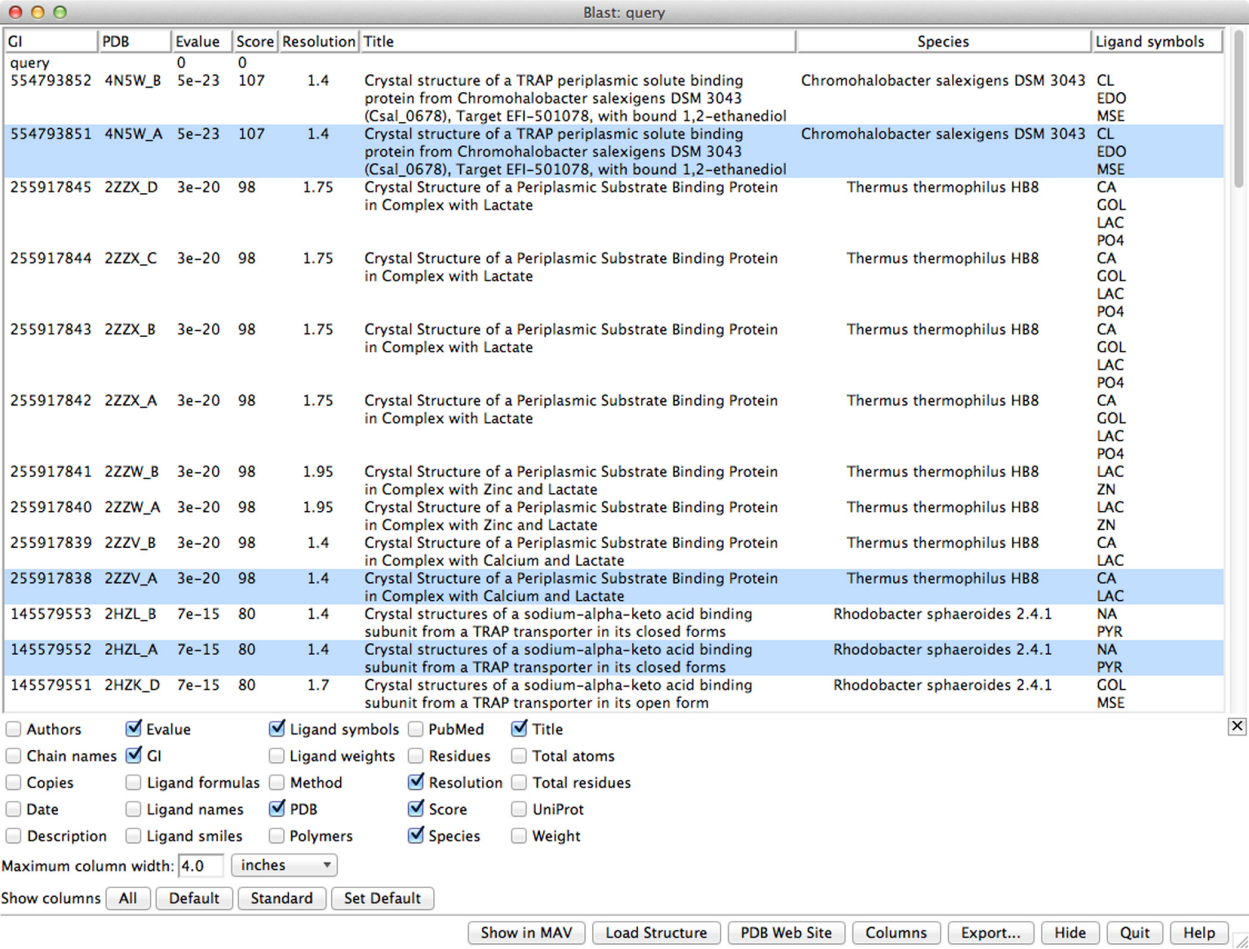
Chimera dialog showing results from BLAST-searching the sequences in the PDB with the query sequence UniProt Q16BX4. The results include multiple chains with the same PDB ID because the searching option to ‘List only best-matching chain per PDB entry’ was unchecked. Additional columns of information such as crystallographic resolution have been fetched from the RCSB PDB.

In Chimera, the sequence of the target can be used to BLAST-search (10) the PDB for known structures that could be used as templates for comparative modeling. The target sequence can be fetched directly from UniProt or simply pasted as plain text into the Blast Protein dialog (‘Tools… Sequence… Blast Protein’ in the main Chimera menu). The RBVI Basic Local Alignment Search Tool (BLAST) service runs the search and returns a list of hits to Chimera. In this example, the option to ‘List only best-matching chain per PDB entry’ was unchecked so that multiple chains in the same structure would be listed as separate hits. The initial output includes BLAST scores and truncated titles, but additional data associated with PDB entries such as crystallographic resolution, full title, ligand names and source species can be interactively retrieved and shown in the dialog using a web service provided by the RCSB PDB (Figure 1). In this case, for modeling the closed form of the target, we want to use high-resolution, ligand-bound structures as templates. Top-scoring PDB entries that fulfill these criteria include 4N5W, 2ZZV (20), 2HZL (21), 4MCO and 2VPN (22). Although some of these proteins exist as dimers, each monomer contains one binding site, and so we choose a monomer (chain A) from each structure as a potential template.

Once the hits of interest have been chosen in the BLAST dialog, their sequences and structures can be loaded into Chimera. Structures are fetched from the RCSB PDB, and the BLAST pseudo-MSA is shown in Chimera's Multalign Viewer. Rather than a true MSA, the alignment from BLAST is a collation of the pairwise alignments of each hit with the query. For comparative modeling, the quality of the sequence alignment between the target and each template is crucial. Despite being the top-scoring hits, none of our chosen templates has >30% sequence identity with the target, or with any other potential template. Thus, the sequences are relatively difficult to align, and re-aligning them may improve the result. Another RBVI web service can be used for this purpose, this time for MSA with Clustal Omega (8). The sequences can be submitted from Multalign Viewer (‘Edit… Realign Sequences’ in the Multalign Viewer menu), and when the results are returned, the new alignment can be shown in another Multalign Viewer window.

**Figure 2.**
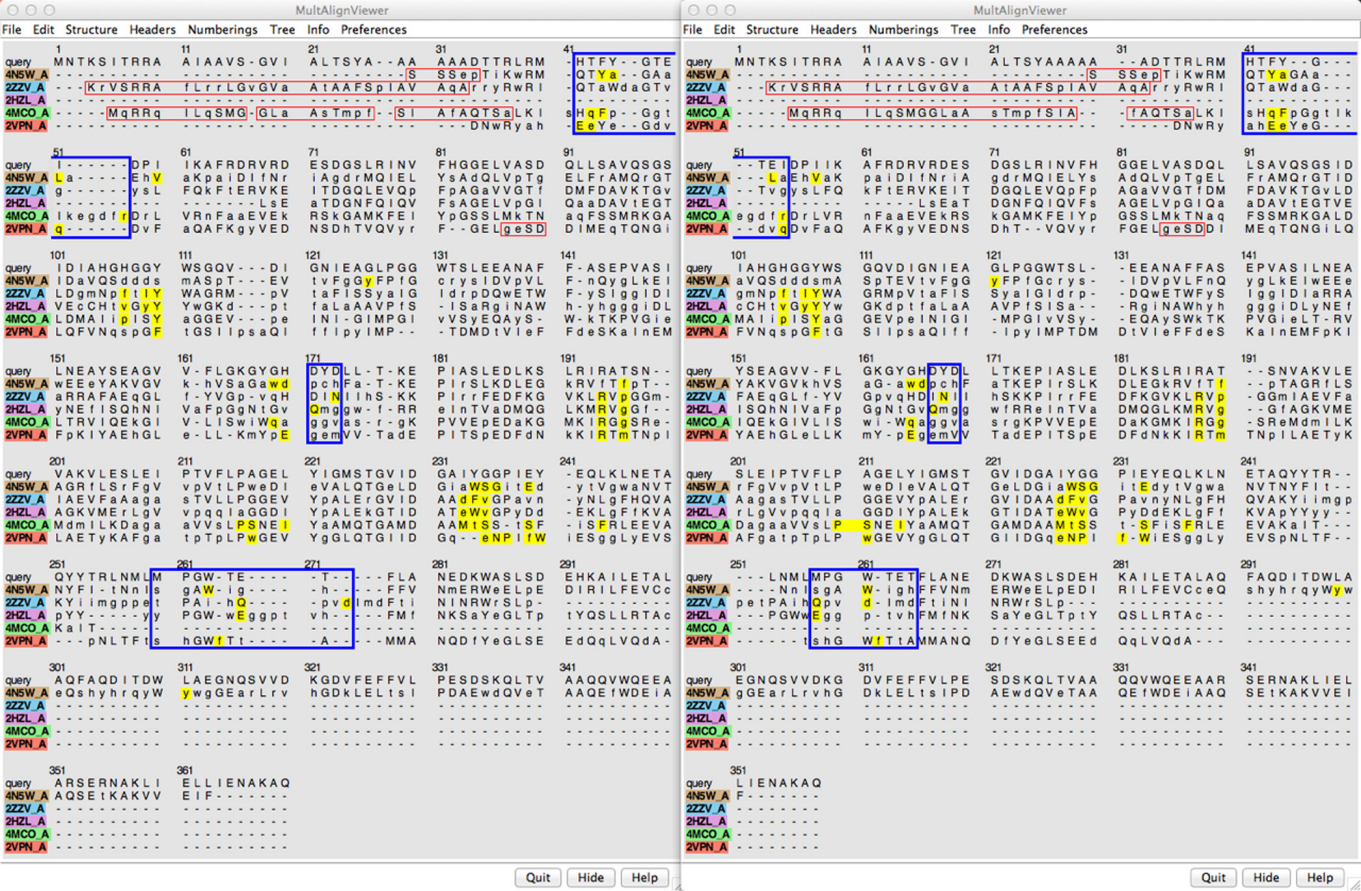
Side-by-side comparison of the BLAST pseudo-MSA (left) and the MSA from Clustal Omega (right) of the query and chains A of 4N5W, 2ZZV, 2HZL, 4MCO and 2VPN. Each alignment is displayed in a Multalign Viewer window in Chimera. Residues within 5 A of the respective bound ligands are highlighted in yellow. Residues missing from the 3D structures are enclosed in red outline boxes. Clustal Omega was run using one iteration and full distance matrices during the initial alignment and iteration. Blue rectangles outline some of the places where binding-site residues in the template structures (yellow) are aligned differently with the query sequence, which would give rise to different binding-site residues in the resulting comparative models.

Differences in the alignments include some reduction of the overall length and slightly more concordance of ligand-proximal residues in the result from Clustal Omega (Figure 2 shows the BLAST alignment on the left and the Clustal Omega alignment on the right; positions within 5 A of the respective ligands are highlighted in yellow). Importantly, there are differences in which target residues are aligned with ligand-proximal template residues; thus, comparative models based on the two alignments would contain different patterns of residues in the presumptive binding site.

**Figure 3.**
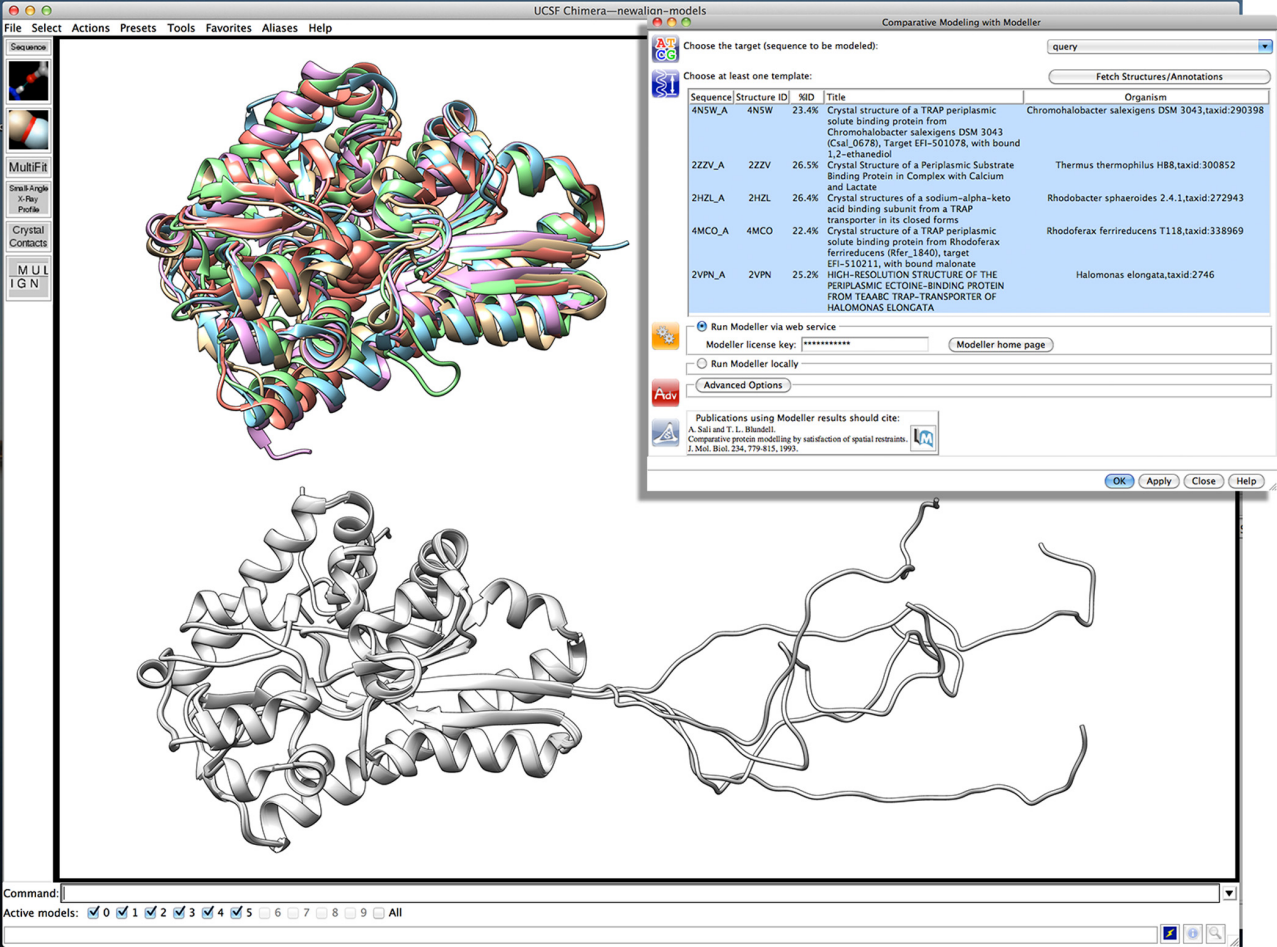
The five template structures (top left), the Modeller input dialog (top right) and the resulting five comparative models (bottom) in Chimera. In the dialog, the query is designated as the target for comparative modeling, and 4N5W, 2ZZV, 2HZL, 4MCO and 2VPN are chosen as the templates. Structures are shown as ribbons, with ligands in the sphere representation. Template structure colors match the boxes around the sequence names in the previous figure, namely: 4N5W in tan, 2ZZV in light blue, 2HZL in light purple, 4MCO in light green and 2VPN in coral. The comparative models are white.

Comparative modeling with Modeller (11), again using an RBVI web service (12), can be launched from the Multalign Viewer window of the desired alignment [‘Structure… Modeller (homology)’ in the Multalign Viewer menu]. The example uses all five of the loaded structures as templates, but any one or more of the structures associated with the sequence alignment could be employed. The user can also specify how many comparative models should be generated. When the calculation is finished, the models are automatically opened in Chimera. Figure 3 shows the template structures (top left), the Modeller input dialog (top right) and five comparative models (bottom). The models are highly similar to one another except for an untemplated segment of about 30 residues at the N-terminus. Another Modeller job could be submitted to refine this segment [via ‘Structure… Modeller (loops/refinement)’ in the Multalign Viewer menu], but it is not near the binding pocket and hence of less interest. Without refinement, examination of the binding pocket could proceed with any of the comparative models.

The biologically relevant ligands of the templates are not necessarily known, but 4N5W has ethanediol in the binding site, 2ZZV has calcium and lactate, 2HZL has sodium and pyruvate [with higher affinity measured for oxovalerate (21)], 4MCO has malonate and 2VPN has ectoine. Of those, only ethanediol lacks a carboxylate group, but it is unlikely to be the physiological ligand. Based on sequence and (model) structure comparisons, the target protein includes the conserved arginine and tyrosine residues that bind the ligand carboxylate group, but may lack the nearby cation-binding site seen in some of the templates (Supplementary Figure S1). Further analyses with Chimera could entail examination of pocket shape and size, hydrophobicity and electrostatic potential (possibly using PDB2PQR and APBS web services provided by the NBCR; see Table 1), and modeling candidate ligands into the site.

## WEB SERVICE INTEGRATION AND IMPLEMENTATION WITH CHIMERA

The Chimera web services described above are made possible by the Opal Toolkit (7) from the NBCR. Chimera bundles and uses the Opal client library to access web services provided by RBVI, which are implemented using the Opal Toolkit server (version 2.5 with Apache Tomcat 6.0). A dashboard (http://webservices.rbvi.ucsf.edu/opal2/dashboard) provides information about the RBVI hosting server and usage statistics, lists available services and provides web interfaces for invoking services directly from the browser. Web services from NBCR have an analogous dashboard (http://nbcr-222.ucsd.edu/opal2/dashboard). Table 1 contains a partial list of the computational web services used by Chimera along with their hosting institutions.

Web services such as those listed in Table 1 may be accessed programmatically using the Opal Toolkit client Web Services Description Language (WSDL) API. WSDL libraries are available for several languages such as Perl, Java and Python. The Opal Toolkit distribution provides both code (http://sourceforge.net/projects/opaltoolkit/files/opal-python/) and documentation (http://nbcr.ucsd.edu/data/docs/opal/docs/2.X/opal-py-index.html) for accessing Opal servers using Python. Supplementary Figure S2 shows a short transcript of how to install the Opal Python client software and to use the generic Opal client to access the RBVI Clustal Omega service (http://webservices.rbvi.ucsf.edu/opal2/CreateSubmissionForm.do?serviceURL=http://localhost:8080/opal2/services%2FClustalOmegaService) on a Linux system. A typical session consists of the following steps.

- Send a ‘launchJob’ request to initiate the computation. The results from the request are a URL and an identifier that are used for monitoring and managing the job.
- Send ‘queryStatus’ requests until the job is finished. The result from a ‘queryStatus’ request may be ‘running’, ‘complete’ or ‘error’. The interval between status query requests is at the discretion of the user. The server will keep job results for several days, so one can either check continuously (typical for short running jobs) or sporadically (for long running jobs).
- Send a ‘getOutputs’ request to get a list of output files. The result from the request is a list of file names and their corresponding URLs. This step may be used whether the job completed successfully (to get computation results) or not (to get error messages).
- Retrieve output files using the URLs obtained in the previous step.

These steps may be executed individually using the generic Opal client or combined into a single Python script. Supplementary Figure S3-A is a Python program that runs the same Clustal Omega alignment computation as shown in Supplementary Figure S2; the output from the program is shown in Supplementary Figure S3-B.

As with the generic Opal client, Chimera also uses the standard Opal client API for communicating with Opal servers (see Supplementary Figure S4). However, unlike the generic Opal client or even the dashboard web interface, Chimera enables the user to invoke the web services in a more facile manner. Rather than having to think about where data files reside, how to convert them into the proper input format for web service input and how often to check job status, Chimera users can invoke tools that allow them to select molecules or sequences as inputs and display results in useful contexts such as interactive 3D models. For example, to recompute an MSA using Clustal Omega, as described in the example above, the user simply opens the MSA in Multalign Viewer and clicks the ‘Edit/Realign Sequences’ menu item; Chimera will then:

- automatically generate the input sequences from the MSA and launch the web service;
- query the server for job status at regular intervals;
- when the job is complete, fetch the output from Clustal Omega, which is a FASTA format MSA file; and
- display the alignment in another Multalign Viewer instance.

For a long-running job, step (ii) may take a long time. While the job is executing, users can save a session, exit from Chimera and restart Chimera at a later time; Chimera keeps track of the web service information in session files and will resume monitoring the outstanding requests. By streamlining the entire job submission, monitoring and retrieval process, Chimera makes it simpler for users to focus on the science rather than data manipulation.

## ACCESS AND LIMITATIONS

- UCSF Chimera is free for non-commercial use. The Chimera Web site, which contains a number of tutorials, documentation and example images and animations may be found at: http://rbvi.ucsf.edu/chimera/. UCSF Chimera is available for Linux, Mac OS X and Windows desktops and users may download released versions as well as nightly development versions. All of the features described above are available in Chimera version 1.9.
- Modeller is free for non-commercial and non-government use and may be downloaded from http://salilab.org/modeller/.
- All web services discussed in this article are freely available with the exception of the Modeller web service, which requires a separate Modeller license.

## SUPPLEMENTARY DATA

Supplementary Data are available at NAR Online.

Supplementary Data
