# Supplementary material for: Enhancing UCSF Chimera through web services
Source: Nucleic Acids Res. 2014 May 26;42(Web Server issue):W478–84. doi: 10.1093/nar/gku377 (PMC4086125; doi:10.1093/nar/gku377)
Supplement: Supplementary Data [file supp_42_W1_W478__index.html]

Supplementary Data 

# Enhancing UCSF Chimera through web services

## Supplementary Data

**Files in this Data Supplement:**

- Supplemental Figures
