# Supplementary material for: Enhancing UCSF Chimera through web services
Source: Nucleic Acids Res. 2014 May 26;42(Web Server issue):W478–84. doi: 10.1093/nar/gku377 (PMC4086125; doi:10.1093/nar/gku377)
Supplement: Supplementary Data [file supp_gku377_nar-00226-web-b-2014-File005.pdf]

SUPPLEMENTARY MATERIALS

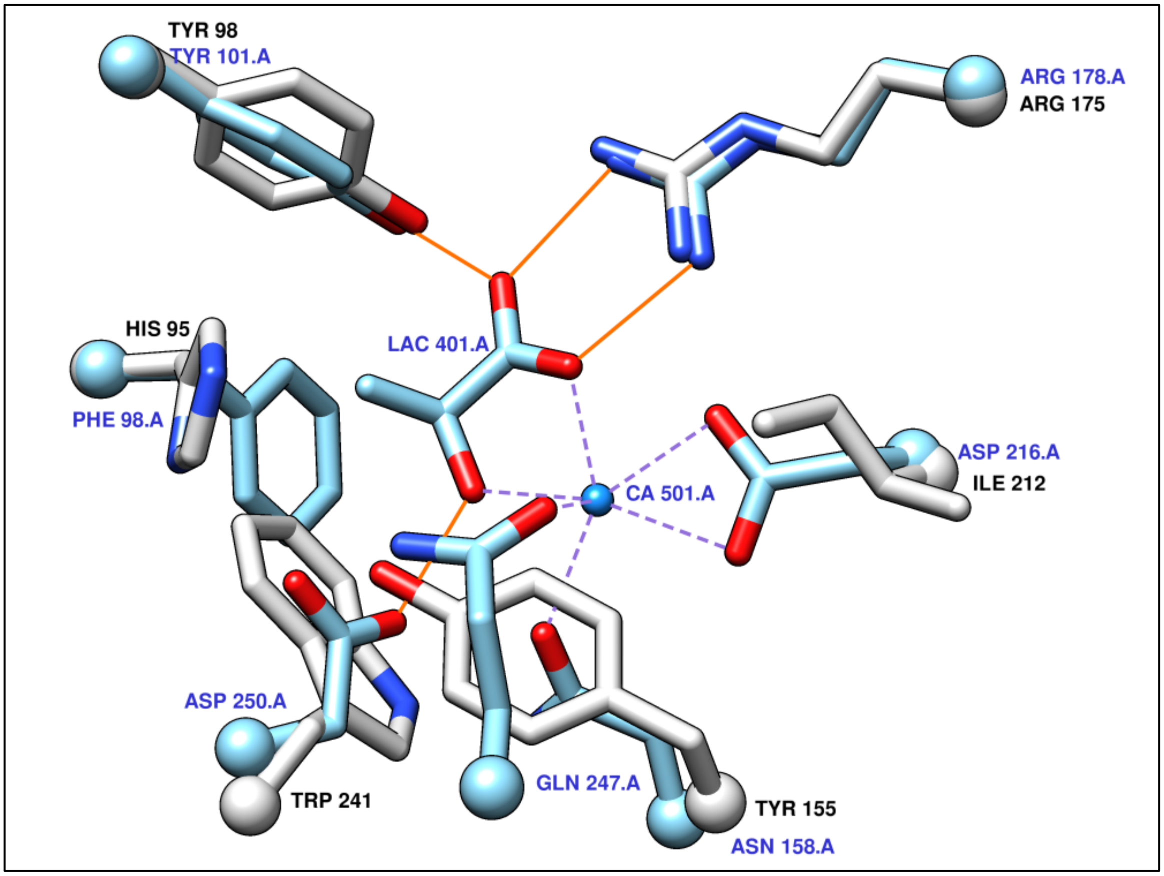

Supplementary Figure S1. Comparison of binding-site residues in the modeled target versus the template 2ZZV using Chimera. Sidechains in a representative comparative model of the target are shown with white carbons, black labels, and standard heteroatom color-coding (red oxygens and blue nitrogens). The structure of 2ZZV includes bound Ca++ and lactate, and is shown with light blue carbons, blue labels, and standard heteroatom color-coding. Hydrogen bonds are shown with solid orange lines, metal-coordination bonds with dashed purple lines. The comparative model includes the tyrosine (Tyr-98) and arginine (Arg-175) residues observed to bind ligand carboxylate, but lacks obvious equivalents to the sidechains that form the ion-binding site in 2ZZV (Asn-158, Asp-216, and Gln-247).

```
$ pwd
/tmp/opal_demo

# Fetch the Opal2 client
$ wget -q http://sourceforge.net/projects/opaltoolkit/ files/opal-python/2.4/opal-py-2.4.1.tar.gz/download
$ ls
3fx2_1ja1_1dx9.fa*  opal-py-2.4.1.tar.gz  typescript

# Unzip the client and add the ZSI egg to your path
$ tar zxf opal-py-2.4.1.tar.gz
$ ls
3fx2_1ja1_1dx9.fa*  opal-py-2.4.1/  opal-py-2.4.1.tar.gz  typescript
$ export PYTHONPATH=$PWD/opal-py-2.4.1/prereqs/ZSI-2.1 _a2-py2.6.egg
$ cd opal-py-2.4.1
/tmp/opal_demo/opal-py-2.4.1
$ ls
ApbsClient.py             CHANGELOG  GenericServiceClient.py  prereqs/
AppService_client.py  docs/    LICENSE              README
AppService_types.py   etc/     OpalClient.py                  wsdl/

# This example uses a FASTA format file with three
# sequences that we want to align
$ cat 3fx2_1ja1_1dx9.fa
>3fx2.chain_A
AKALIVYGSTTGNTEYTAETIARELADAGYEVDSRDAASVEAGGLFE----GFDLVLLGC
STWGD-DSIELQDDFIPLFDSLEETGAQ--GRKVACFGCGDS--SYEYFCGAVDAIEEKL
KNLGAEIV----QDGLRIDGDPRAARD-DIVGWAHDVRGAI

>1JA1_B
---IVfYGSqTGTAEefANrLSKDahryGmRgmSaDpeEyDlAdLsSlpeidkSLVVFcm
ATYGEgDptDnaQDF---YDwLQETdVDltGvKfAvFGlGNK--TYEHFnAmgkyVDQRL
EQLGAQrI----fE-LgLgdDdgnlEE-DFItW--------

>1DX9_D
----LfYGTqTGKTEsVAEiIrdEFGNd--vVtlHDVSQAEVtdLnD-----YQyLIIGC
pT--a-NigELQSDWegLYSELDDVdfN--GKlVAyFGtGDQigyaDnFqdAIgiLEEKI
SQrGGKtVgywstDGydFN-DsKAlRNgkFVGlAlD-----

# Copy in the file
$ cp ../3fx2_1ja1_1dx9.fa .

# Execute the generic client
$ python GenericServiceClient.py -l http://webserviices.rbvi.ucsf.edu/opal2/services/ClustalOmegaService -r launchJob -a
"-i 3fx2_1jja1_1dx9.fa -o msa.fa" -f 3fx2_1ja1_1dx9.fa

Launching remote ClustalOmegaService job
Received Job ID: appClustalOmegaService1389737965922556835590
Base Output URL: http://webservices.cgl.ucsf.edu/appClustalOmegaService1389737965922556835590
$ python GenericServiceClient.py -l http://webservices.rbvi.ucsf.edu/opal2/services/ClustalOmegaService -r queryStatus -
j appClustalOmegaService1389737965922556835590
ClustalOmegaService status:
          Code: 8
          Message: Execution complete - check outputs to verify successful execution
          Output Base URL: http://webservices.cgl.ucsf.edu/appClustalOmegaService1389737965922556835590
$ python GenericServiceClient.py -l http://webserviices.rbvi.ucsf.edu/opal2/services/ClustalOmegaService -r getOutputs -
j appClustalOmegaService1389737965922556835590
Retrieving ClustalOmegaService status
Job ClustalOmegaService appClustalOmegaService1389737965922556835590 execution terminated successfully
          Code: 8
          Message: Execution complete - check outputs to verify successful execution
          Output Base URL: http://webservices.cgl.ucsf.edu/appClustalOmegaService1389737965922556835590
          Standard Output: http://webservices.cgl.ucsf.edu/appClustalOmegaService1389737965922556835590/stdout.txt
          Standard Error: http://webservices.cgl.ucsf.edu/appClustalOmegaService1389737965922556835590/stderr.txt
          http://webservices.cgl.ucsf.edu/appClustalOmegaService1389737965922556835590/msa.fa
          http://webservices.cgl.ucsf.edu/appClustalOmegaService1389737965922556835590/3fx2_1ja1_1dx9.fa

# Fetch the results
$ wget -q http://webservices.cgl.ucsf.edu/appClustalOmegaService1389737965922556835590/msa.fa
$ cat msa.fa
>3fx2.chain_A
AKALIVYGSTTGNTEYTAETIARELADAGYEVDSRDAASVEAGGLFE----GFDLVLLGC
STWGD-DSIELQDDFIPLFDSLEETG--AQGRKVACFGCGDS--SYEYFCGAVDAIEEKL
KNLGAEIV----QDGLRIDGDPRAARDDIVGWAHDVRGAI
>1JA1_B
---IVfYGSqTGTAEefANrLSKDahryGmRgmSaDpeEyDlAdLsSlpeidkSLVVFcm
ATYGEgDptDnaQDF---YDwLQETdVDltGvKfAvFGlGNK--TYEHFnAmgkyVDQRL
EQLGAQrI-----fELgLgdDdgnlEEDFItW--------
>1DX9_D
----LfYGTqTGKTEsVAEiIrdEFGNd--vVtlHDVSQAEVtdLn-----DYQyLIIGC
pT--a-NigELQSDWegLYSELDDVd--fNGKlVAyFGtGDQigyaDnFqdAIgiLEEKI
SQrGGKtVgywstDGydFNDsKAlRNgkFVGlAlD-----
$ exit
```

Supplementary Figure S2. Example session showing how to download and execute the Opal Generic Client to access the RBVI Clustal Omega web service.

```
#
# This sample program runs a Clustal Omega job via the RBVI web service.
#
# In order to run this script, you need to install the Opal Python client
# (see http://nbcr.ucsd.edu/data/docs/opal/docs/2.X/opal-py-index.html).
# Follow the installation instructions, including setting the PYTHONPATH
# environment variable if necessary.  Place this file (sample.py) in the
# opal-py-2.4.1 directory.  From that directory, you should then be able
# to execute:
#
#    python sample.py
#
# to run the Clustal Omega job.
#

seqData = """>3fx2.chain_A
AKALIVYGSTTGNTEYTAETIARELADAGYEVDSRDAASVEAGGLFE----GFDLVLLGC
STWGD-DSIELQDDFIPLFDSLEETGAQ--GRKVACFGCGDS--SYEYFCGAVDAIEEKL
KNLGAEIV----QDGLRIDGDPRAARD-DIVGWAHDVRGAI

>1JA1_B
---IVfYGSqTGTAEefANrLSKDahryGmRgmSaDpeEyDlAdLsSlpeidkSLVVFcm
ATYGEgDptDnaQDF---YDwLQETdVDltGvKfAvFGlGNK--TYEHFnAmgkyVDQRL
EQLGAQrI----fE-LgLgdDdgnlEE-DFItW--------

>1DX9_D
----LfYGTqTGKTEsVAEiIrdEFGNd--vVtlHDVSQAEVtdLnD-----YQyLIIGC
pT--a-NigELQSDWegLYSELDDVdfN--GKlVAyFGtGDQigyaDnFqdAIgiLEEKI
SQrGGKtVgywstDGydFN-DsKAlRNgkFVGlAlD-----
"""

import OpalClient

# Service URL
URL = "http://webservices.rbvi.ucsf.edu/opal2/services/ClustalOmegaService"
client = OpalClient.OpalService(URL)

# command-line arguments and input data file
argList = "-i input.fa -o msa.fa"
with open("input.fa", "w") as f:
   f.write(seqData)
inputFile = [ "input.fa" ]

print "Launching remote Clustal Omega job"
jobStatus = client.launchJobNB(argList, inputFile)

import time
while jobStatus.isRunning() :
   time.sleep(3)
   print "Polling job status"
   jobStatus.updateStatus()

if jobStatus.isSuccessful():
   print "Job execution finished sucessfully."
else:
   print "Job execution failed."
   print "Check the stdout.txt and the stderr.txt for errors...."

def show_url(url):
   import os.path
   print "%s:" % os.path.basename(url)
   import urllib
   filename, headers = urllib.urlretrieve(url)
   with open(filename) as f:
      print f.read()

show_url(jobStatus.getURLstdout())
show_url(jobStatus.getURLstderr())
for url in jobStatus.getOutputFiles():
   show_url(url)
```

Supplementary Figure S3-A. Sample Python code for accessing Clustal Omega using RBVI's Web Services.   Note the instructions for execution in the header.

```
$ python sample.py
Launching remote Clustal Omega job
Polling job status
Job execution finished sucessfully.
stdout.txt:

stderr.txt:

msa.fa:
>3fx2.chain_A
AKALIVYGSTTGNTEYTAETIARELADAGYEVDSRDAASVEAGGLFE----GFDLVLLGC
STWGD-DSIELQDDFIPLFDSLEETG--AQGRKVACFGCGDS--SYEYFCGAVDAIEEKL
KNLGAEIV----QDGLRIDGDPRAARDDIVGWAHDVRGAI
>1JA1_B
---IVfYGSqTGTAEefANrLSKDahryGmRgmSaDpeEyDlAdLsSlpeidkSLVVFcm
ATYGEgDptDnaQDF---YDwLQETdVDltGvKfAvFGlGNK--TYEHFnAmgkyVDQRL
EQLGAQrI-----fELgLgdDdgnlEEDFItW--------
>1DX9_D
----LfYGTqTGKTEsVAEiIrdEFGNd--vVtlHDVSQAEVtdLn-----DYQyLIIGC
pT--a-NigELQSDWegLYSELDDVd--fNGKlVAyFGtGDQigyaDnFqdAIgiLEEKI
SQrGGKtVgywstDGydFNDsKAlRNgkFVGlAlD-----

input.fa:
>3fx2.chain_A
AKALIVYGSTTGNTEYTAETIARELADAGYEVDSRDAASVEAGGLFE----GFDLVLLGC
STWGD-DSIELQDDFIPLFDSLEETGAQ--GRKVACFGCGDS--SYEYFCGAVDAIEEKL
KNLGAEIV----QDGLRIDGDPRAARD-DIVGWAHDVRGAI

>1JA1_B
---IVfYGSqTGTAEefANrLSKDahryGmRgmSaDpeEyDlAdLsSlpeidkSLVVFcm
ATYGEgDptDnaQDF---YDwLQETdVDltGvKfAvFGlGNK--TYEHFnAmgkyVDQRL
EQLGAQrI----fE-LgLgdDdgnlEE-DFItW--------

>1DX9_D
----LfYGTqTGKTEsVAEiIrdEFGNd--vVtlHDVSQAEVtdLnD-----YQyLIIGC
pT--a-NigELQSDWegLYSELDDVdfN--GKlVAyFGtGDQigyaDnFqdAIgiLEEKI
SQrGGKtVgywstDGydFN-DsKAlRNgkFVGlAlD-----

$ exit
```

Supplementary Figure S3-B. Output from the Python program shown in Figure S3A.

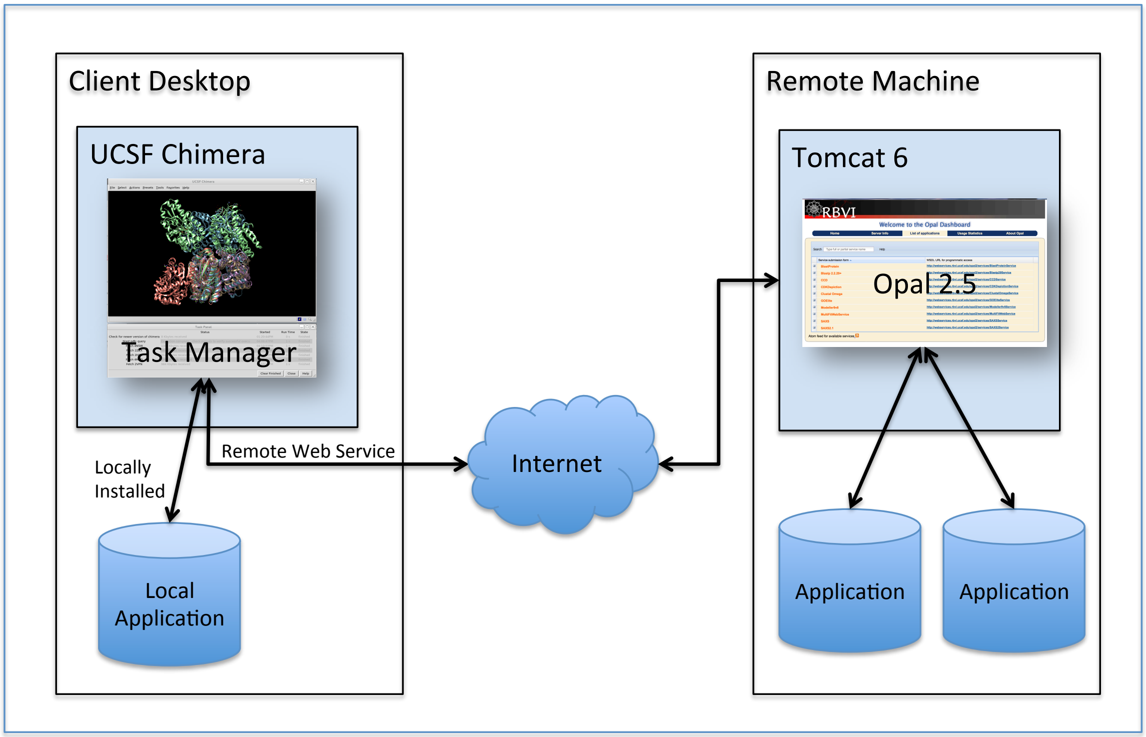

Supplementary Figure S4. Chimera Web Services architecture. UCSF Chimera communicates via the Internet with remote hosts that run the Opal Toolkit on an Apache Tomcat server. Chimera can launch a web service request and monitor its progress using the Task Manager; when the request completes, the Task Manager notifies Chimera and the results are processed and displayed. Service requests can also be run locally if the required applications are installed. The existence of long running, computational intensive jobs is saved in Chimera session files and the monitoring of these jobs can resume when Chimera is restarted at a later time.
